# MR image‐based synthetic CT for IMRT prostate treatment planning and CBCT image‐guided localization

**DOI:** 10.1120/jacmp.v17i3.6065

**Published:** 2016-05-08

**Authors:** Shupeng Chen, Hong Quan, An Qin, Seonghwan Yee, Di Yan

**Affiliations:** ^1^ Laboratory of Biological & Medical Physics and Key Laboratory of Artificial Micro‐ & Nano‐ structures of Ministry of Education School of Physics and Technology, Wuhan University Wuhan China; ^2^ Department of Radiation Oncology Beaumont Health System Royal Oak MI USA

**Keywords:** MRI, radiotherapy, synthetic CT, deformable image registration, autosegmentation, CBCT

## Abstract

The purpose of this study was to propose and evaluate a method of creating a synthetic CT (S‐CT) from MRI simulation for dose calculation and daily CBCT localization. A pair of MR and CT images was obtained in the same day from each of 10 prostate patients. The pair of MR and CT images was preregistered using the deformable image registration (DIR). Using the corresponding displacement vector field (atlas‐DVF), the CT image was deformed to the MR image to create an atlas MR‐CT pair. Regions of interest (ROI) on the atlas MR‐CT pair were delineated and used to create atlas‐ROI masks. ‘Leave‐one‐out’ test (one pair of MR and CT was used as subject‐MR and subject‐CT for evaluation, and the remaining 9 pairs were in the atlas library) was performed. For a subject‐MR, autosegmentation and DVFs were generated using DIR between the subject‐MR and the 9 atlas‐MRs. An S‐CT was then generated using the corresponding 9 paired atlas‐CTs, the 9 atlas‐DVFs and the corresponding atlas‐ROI masks. The total 10 S‐CTs were evaluated using the Hounsfield unit (HU), the calculated dose distribution, and the auto bony registration to daily CBCT images with respect to the 10 subject‐CTs. HU differences (mean±STD) were (2.4±25.23), (1.18±39.49), (32.46±81.9), (0.23±40.13), and (3.74±144.76) for prostate, bladder, rectal wall, soft tissue outside all ROIs, and bone, respectively. The discrepancy of dose‐volume parameters calculated using the S‐CT for treatment planning was small (≤0.22% with 95% confidence). Gamma pass rate (2% & 2 mm) was higher than 99.86% inside PTV and 98.45% inside normal structures. Using the 10 S‐CTs as the reference CT for daily CBCT localization achieved the similar results compared to using the subject‐CT. The translational vector differences were within 1.08 mm (0.37±0.23 mm), and the rotational differences were within 1.1° in all three directions. S‐CT created from a simulation MR image using the proposed approach with the preconstructed atlas library can replace the planning CT for dose calculation and daily CBCT image guidance.

PACS number(s): 87.57.nf, 87.57.nm

## I. INTRODUCTION

MR image has been widely used in radiation therapy for organ and tumor delineation due to its superior soft‐tissue contrast. Previous studies have shown that substantial improvement of accuracy in target and normal organs delineation can be achieved via using MR images.^1^, ^2^, ^3^

In the current clinic practice, however, MRI is used as a secondary image for ROI delineation after a rigid body registration to the simulation CT image. The CT image is used for the treatment planning dose calculation and as the reference image in daily CBCT image guidance. This process unavoidably suffers from uncertainty caused by the MR‐CT image registration. The uncertainty of rigid registration between prostate CT and MR was about 2 mm (STD) evaluated by implanted seeds.^(4)^ These uncertainties may produce a systematic shift in the delineation/propagation and lead to dose discrepancy ultimately.^(5)^

MRI stand‐alone radiotherapy has the ability to eliminate image registration uncertainty and reduce clinical workload. Clinical implementation of the MRI‐alone radiotherapy relies on a method to derive CT‐equivalent information from MR image for dose calculation in treatment planning and treatment position localization. Such MR‐based CT‐equivalent data has been referred to the substitute CT^(6)^ or S‐CT. Different methods for generating S‐CT have been proposed. For image intensity/segmentation‐based conversion methods,^7^, ^8^, ^9^, ^10^, ^11^ specific MR sequences, such as the ultrashort echo time (UTE)^10^, ^11^ sequence, were used to achieve accurate separation of air and bony tissue for creating S‐CT. While providing promising results, they are not practical since these special sequences are not generally available in clinic. DIR‐based methods have also been explored to generate S‐CT.^12^, ^13^, ^14^, ^15^, ^16^ However, using DIR algorithms directly^12^, ^13^ to map HU from the CT images in the atlas library to an S‐CT image suffers from inherent registration errors due to large anatomical differences between different patients. It is possible to reduce registration uncertainty, either by improving the accuracy of DIR algorithm or by combining with a “CT voxel intensity selection algorithm”.^14^, ^16^ Dosimetric aspect of using MR image‐derived S‐CT image in radiotherapy treatment planning has been well investigated. In addition, it would be of particular value to examine feasibility of using S‐CT as the reference image for online CBCT image‐guided radiotherapy (IGRT).

In this study, we proposed a novel method for S‐CT image voxel intensity selection based on multi‐atlas‐based DIR with a specifically constructed atlas library. This specific atlas library contains multiple atlas MR‐CT paired images, atlas‐DVF, and atlas‐ROI masks. The major differences of this method to those previously published are the construction of the atlas library and the “selection algorithm”. The method was evaluated systematically based on the accuracy of the autosegmentation, the HU difference, the dose calculation for treatment planning, and the target localization for online CBCT image‐guided treatment.

## II. MATERIALS AND METHODS

### A. Atlas library construction

Ten MR‐CT paired images, as well as organs of interest, of prostate cancer patients who received radiotherapy in our institution from November 2013 to November 2014 were used in the study. Pretreatment CT (P‐CT) image and MR image of each of 10 prostate patients acquired at the similar time of the treatment simulation day were selected to be the paired images. No significant change of major anatomies was observed between the two different modality images. P‐CT images were acquired using a Brilliance Big Bore (Philips Healthcare, Cleveland, OH) scanner with a reconstructed pixel size $\approx 1.12\times 1.12\times 3\;{\text{mm}}^{3}$. The MR images (T2‐weighted) were acquired using a 3.0‐T Ingenia (Philips Healthcare) high‐field system with spin‐echo sequence (T2‐TSE), TE/TR≈110/5000 ms, flip angle=90°, and pixel size $\approx 0.45\times 0.45\times 3\;{\text{mm}}^{3}$. P‐CT image was used for the planning dose calculation, and also the reference image for daily CBCT image‐guided treatment localization and correction.

In order to generate better ‘matched’ paired MR‐CT images, an efficient inverse‐consistent diffeomorphic image registration method with normalized mutual information (NMI) algorithm (ADMIRE, CMS Software, Elekta Inc., Stockholm, Sweden) was used for intrapatient DIR between the MR and CT paired images.^(17)^ An atlas‐CT image was then generated by applying the corresponding DVF (atlas‐DVF) from the P‐CT image to the corresponding MR image.

**Figure 1:**
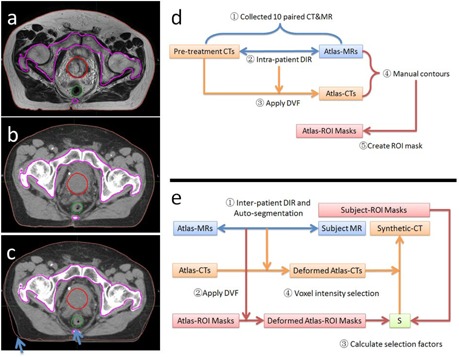
A representative (a) atlas‐MR, (b) atlas‐CT, and (c) P‐CT with (d) flow charts of atlas library construction and (e) S‐CT generation. P‐CT = pretreatment CT image; DIR = deformable image registration; DVF = displacement vector field.

The MR image (atlas‐MR image) and the atlas‐CT image formed a paired atlas MR‐CT which was matched voxel‐by‐voxel and shared all regions of interest (ROI). The ROIs included bladder, prostate and rectum delineated manually on the atlas‐MR image, and skin contours, air and bone delineated semiautomatically on the atlas‐CT image based on image‐intensity threshold. A representative atlas MR‐CT paired image with the corresponding P‐CT is shown in Figs. 1(a), (b) and (c).

Binary masks of ROIs on each atlas MR‐CT were created based on the corresponding contours. Each atlas‐ROI mask is a three‐dimension matrix with voxels labeled 1 inside and 0 outside the ROI contour, such as:

(1) $$atlas\_mask_{i,j}(p)=\{\begin{array}{ll} 1 & if\;p\in C_{i,j}\\ 0 & if\;p\notin C_{i,j} \end{array}$$

where *i* is the index of atlas paired images, *j* is the index of ROI; *atlas_mask_i,j_* is the j‐th ROI defined on the i‐th atlas paired images, and *C_i,j_* is j‐th ROI on the i‐th atlas; *p* is the image voxel position. The atlas library includes all paired atlas‐MR and atlas‐CT images, as well as the corresponding atlas‐ROI masks. Six ROI masks (air, bone, bladder, prostate, rectum, and skin) of each patient are included. The flow chart of atlas library construction is illustrated as Fig. 1(d).

### B. S‐CT generation

#### B.1 MRI DIR and autosegmentation

Utilizing the atlas library, the multi‐atlas‐based DIR^(16)^ (ADMIRE, CMS Software, Elekta Inc.) was performed between a subject‐MR image and the atlas‐MR images. Intensity‐weighted multi‐atlas label fusion method^18^, ^19^, ^20^ was used for ROI autosegmentation (bone, prostate, rectum, and bladder) on the subject‐MR image. Auto‐segmentation of soft‐tissue ROIs (bladder, rectum and prostate) were checked visually and manually corrected if autosegmentation was unacceptable. Air in rectum was delineated semiautomatically based on the threshold of MR image intensity. And skin contour was generated automatically using the whole body function. ROI masks‐(MIM, Software, Inc., Cleveland, OH., US.) on the subject‐MR were then created based on the resulting contours:

(2) $$subject\_mask_{j}(p)=\{\begin{array}{ll} 1 & if\;p\in C_{j}\\ 0 & if\;p\notin C_{j} \end{array}$$

where *subject_mask_j_* is the j‐th ROI mask, and *C_j_* is j‐th ROI defined on the subject‐MR image.

The resulting DVF from the subject‐MR image to the i‐th atlas‐MR image was indicated as

(3) $${\vec{d}}_{l}=\varphi (atlas\_MR_{i},\;subject\_MR)$$

The DVFs were applied to the corresponding atlas‐CT images and atlas‐ROI masks to generate the deformed atlas‐CTs and deformed atlas‐ROI masks on the subject‐MR image space:

(4) $$def\_atlas\_CT_{i}(p)=atlas\_CT_{i}(p+{\vec{d}}_{l}(p))$$

(5) $$def\_atlas\_mask_{i,j}(p)=atlas\_mask_{i,j}(p+{\vec{d}}_{l}(p))$$

where *def_atlas_CT_i_* is the deformed atlas‐CT image generated from i‐th atlas‐CT image, and *def_atlas_mask_i,j_* is the deformed atlas‐ROI mask generated form j‐th atlas‐ROI mask on i‐th atlas‐CT.

#### B.2 S‐CT voxel intensity selection

If a voxel is located inside a ROI on the subject‐MR image, and its corresponding position on i‐th atlas MR‐CT images is also inside the same ROI, then the voxel intensity value from i‐th atlas‐CT can be selected to determine the voxel intensity on the S‐CT.

In our selection algorithm, a selection factor used for atlas selection based on the intersection of atlas‐ROI masks is determined by the following formula:

(6) $$w_{i,j}(p)=\{\begin{array}{ll} 1 & if\;subject\_mask_{j}(p)=def\_atlas\_mask_{i,j}(p)\\ 0 & f\;subject\_mask_{j}(p)\neq def\_atlas\_mask_{i,j}(p) \end{array}$$

For each atlas,

(7) $$w_{i}(p)=\prod_{j}w_{i,j}(p)$$

where *i* is the index of atlas paired images, *j* is the index of ROIs; $w_{i,j}(p)$ is the selection factor calculated based on the intersection of j‐th ROI between the subject‐ROI mask and i‐th atlas‐ROI mask; $w_{i}(p)$ is the selection factor for i‐th atlas‐CT for voxel position *p.*

The S‐CT image intensity at voxel p is then determined using the arithmetic mean intensity of those ‘selected’ from the deformed atlas‐CT images:

(8) $$S\_CT(p)\{\begin{array}{ll} \frac{1}{\sum_{i=1}^{n}w_{i}(p)}\cdot \sum_{i=1}^{n}w_{i}(p)\cdot def\_atlas\_CT_{i}(p) & if\sum_{i=1}^{n}w_{i}(p)\neq 0\\ \frac{1}{n}\cdot \sum_{i=1}^{n}def\_atlas\_CT_{i}(p) & if\sum_{i=1}^{n}w_{i}(p)=0 \end{array}$$

where *n* is the number of atlas MR‐CT images in the atlas‐library; $\sum_{i=1}^{n}w_{i}(p)\neq 0$ means at least one atlas is ‘selected’, otherwise, use the direct arithmetic mean intensity of all deformed atlas‐CT images as the S‐CT intensity at the voxel *p*.

Air in S‐CT image was assigned −1000 HU based on air mask defined on the subject‐MR image. The flow chart of S‐CT image generation is illustrated as Fig. 1(e).

### C. Evaluation

‘Leave‐one‐out’ test was performed for evaluation. One paired MR‐CT image out of the 10 was used as the subject‐MR image to generate according S‐CT image. The corresponding P‐CT, the predetermined DVF (atlas‐DVF) between the MR image and the P‐CT image, ROIs, as well as the corresponding planned dose distribution and daily CBCT localization, were used to evaluate the quality and accuracy of the S‐CT. A total of 10 S‐CT images were created and evaluated in the study.

The dice similarity coefficient (DSC2[A∩B]/[A+B]) was applied to analyze the performance of the autosegmented ROIs on the subject MR image with respect to those of manually delineated. The HU values inside ROIs on S‐CT image were compared to those on the atlas‐CT image.

IMRT plan (7 beams with dose 79.2 Gy prescribed to PTV) was used for evaluation of dose calculations. Each S‐CT was deformed to the corresponding P‐CT using the predetermined atlas‐DVF between the MR and P‐CT images. Dose distribution was recalculated on the deformed S‐CT, and compared to the one calculated on the P‐CT. Dose grid in the dose calculation included all ROIs with a resolution $\approx 1.5\times 1.5\times 3\;{\text{mm}}^{3}$. Dose distributions were analyzed using 2D local gamma index with different criteria. DVH‐parameters of PTV ($D_{99}$, D_95_) and organs at risk (OARs) ($D_{40},D_{5}$ for rectum, $D_{50}$ for bladder and D1 for femoral region) were compared.

In addition, the deformed S‐CT image replaced the P‐CT as the reference image for daily treatment CBCT image registration for 363 daily CBCTs obtained from the 10 patients' daily treatments. Rigid body (pelvic bone) image registration with six degree of freedom of shift and rotation between the reference and the daily CBCT was performed using the XVI software tool (XVI, Elekta, Inc.). Setup error in XVI is expressed by three translational shifts and three rotational errors along the three reference axes. Both translational and rotational differences between using S‐CT and P‐CT as the reference were evaluated.

## III. RESULTS

### A. Performance of autosegmentation

**Figure 2:**
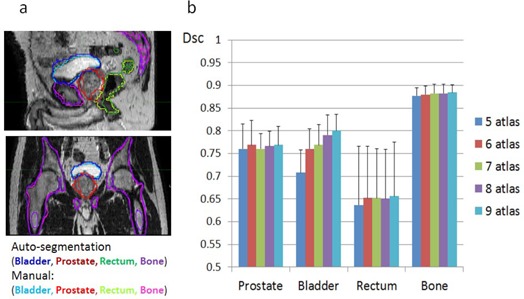
Performance of multi‐atlas autosegmentation of subject‐MR: (a) an average case and (b) statistical results of all 10 subject‐MRs using different number of atlas. DSC = dice similar coefficient.

Figure 2(a) shows the autosegmentation results of an average case. The mean DSC ± STD for all 10 subject‐MR images were (0.77±0.04), (0.8±0.04), (0.66±0.12), and (0.88±0.02) for prostate, bladder, rectum, and bone, respectively. An average case of autosegmentation results was shown in Fig. 2(a). The performance of autosegmentation using different number of atlas (5–9) was summarized in Fig. 2(b). Generally, only the DSC of bladder increases significantly with atlas number. The DSC of rectum is the lowest among four ROIs, stable around 0.65 with atlas number increase from 6 to 9. The autosegmentation gave reasonable initial contours for prostate and bone, with DSC over 0.75 and 0.85, respectively, by all tested atlas numbers.

### B. HU accuracy of S‐CT images

**Figure 3:**
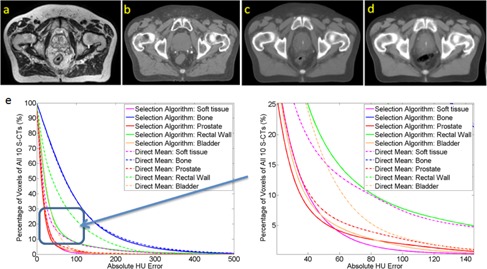
An example patient (a) atlas‐MR, (b) paired atlas‐CT and S‐CT generated via using (c) the proposed method and (d) the direct arithmetic mean method; (e) cumulative histogram of HU absolute error of all voxels from 10 S‐CTs using the proposed method (solid line) and direct arithmetic mean method (dash line). HU = Hounsfield unit.

Figure 3(c) presents a representative S‐CT image created based on the proposed method. The (mean±STD) of HU error for the 10 S‐CTs are (2.4±25.23), (1.18±39.49), (32.46±81.9), (0.23±40.13), and (3.74±144.76) for prostate, bladder, rectal wall, soft tissue outside all ROIs, and bone, respectively. The cumulative histogram of HU absolute error of all voxels among the 10 S‐CTs is shown on Fig. 3(e). Absolute HU errors of 90% voxels of soft tissue were less than 46 HU using the proposed method, and 74 HU using the direct mean method.^12^, ^13^ Eighty percent (80%) of bone voxels had absolute HU errors less than 150 HU using the proposed method, and 153 HU using the direct mean method.

### C. Dose calculation

Overall, the dose distributions calculated on S‐CTs were in good agreement to the original planning dose. The point dose discrepancy for all 10 evaluated patients was small. Gamma pass rate (2% and 2 mm) is higher than 99.98% inside PTV and 98.45% inside normal structures (Table 1). Deviation of DVH‐parameters ($(\text{PTV}D_{99}\;\setminus D_{95}$, $\text{Rectum}D_{40}\setminus D_{5}\%$, and Bladder‐$D_{50}$) for all 10 evaluated plans was less than 1%. Specifically, the deviation of $D_{99}$ and $D_{95}$ of PTV is less than 0.33%; $D_{40}\setminus D_{5}$ of rectum, $D_{50}$ of bladder and $D_{1}$ of femoral region are less than 0.43% (Table 2).

**Table 1:** Results of dose calculation using 10 S‐CTs showing 2D local gamma pass rate.

*2D Local Gamma Pass Rate Mean (STD)*		
ROI\Criteria	1% & 1 mm	2% & 2 mm
PTV	99.98% (0.1%)	100% (0)
Bladder	99.64% (0.01%)	99.98% (0.01%)
Rectum	98.44% (1.41%)	99.64% (0.01%)
Femoral Region	99.16% (0.01%)	99.97% (0.01%)

ROI = regions of interest; 1% & 1 mm = gamma criteria 1% dose discrepancy and 1 millimeter; PTV = planning target volume; STD = standard deviation.

**Table 2:** Results of dose calculation using 10 S‐CTs, showing relative discrepancy of DVH‐parameters.

*Relative Discrepancy of DVH‐Parameters*			
*DVH‐Parameter*	*Mean (STD)*	*Range*	*95% Confidence Interval (t‐distribution)*
$\text{PTV}-D_{99}$	0.11% (0.13%)	(−0.02%–0.33%)	(0, 0.2%)
$\text{PTV}-D_{95}$	0.09% (0.11%)	(−0.04%–0.28%)	(0, 0.17%)
$\text{Bladder}-D_{50}$	0.07% (0.2%)	(−0.23%–0.43%)	(−0.01%,0.21%)
$\text{Rectum}-D_{40}$	0.09% (0.12%)	(−0.06%–0.29%)	(0, 0.18%)
$\text{Rectum}-D_{5}$	0.11% (0.15%)	(−0.1%–0.35%)	(0, 0.22%)
Femoral Region‐D_1_	0.07% (0.15%)	(−0.08%–0.32%)	(0, 0.17%)

PTV = planning target volume; STD = standard deviation; DVH = dose‐volume histogram.

### D. CBCT localization evaluation

**Figure 4:**
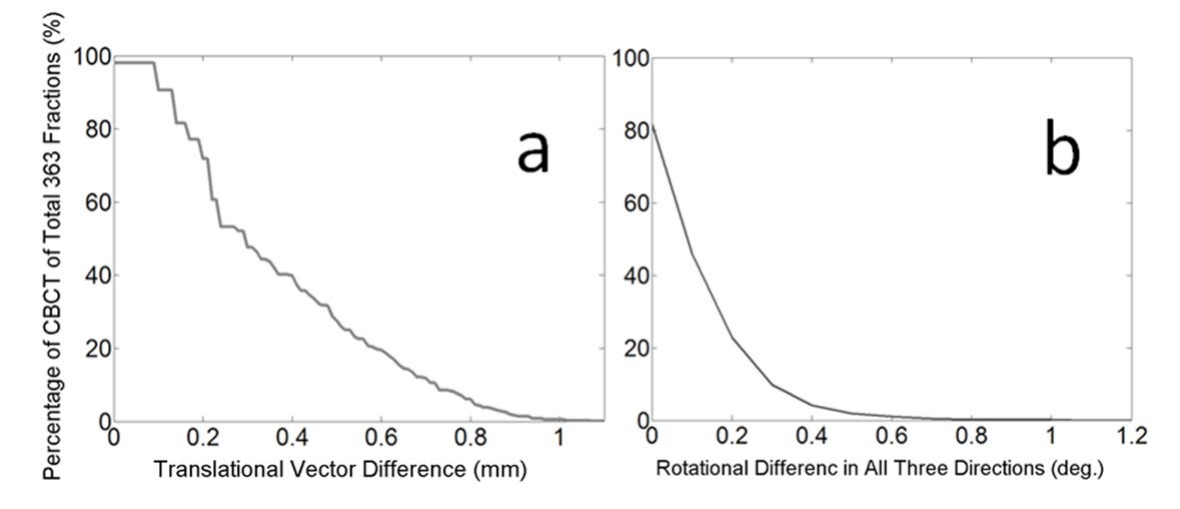
Localization results: cumulative probability distribution of (a) translational difference and (b) rotational difference.

Using registered S‐CTs as reference images for daily CBCT localization achieved similar results compared with those obtained using P‐CTs. Difference of the results are shown on Fig. 4. Translational vector difference is less than 1 mm (99.45%) and 1.08 mm (100%). All rotational differences are smaller, <0.5°(98.07%) and <1.1°(100%).

## IV. DISCUSSION

The proposed method is novel in several aspects. First, multiple MR‐CT ‘matched’ paired images, as well as the ROI masks, were generated using an efficient inverse‐consistent diffeomorphic image registration method,^(17)^ and included in the atlas library for atlas‐based DIR. This is critical, especially for prostate patients, due to visible organ deformations between different modality images even when the images acquired in the same day (Figs. 1(a), (b) and (c)). Second, S‐CT image intensity was selected voxel‐by‐voxel using the predesigned atlas‐ROI masks and the multiple atlas‐CT images. The proposed method can also be easily generalized to different treatment sites with different MR sequences. Third, comprehensive evaluations of the S‐CT images were performed including autosegmentation, HU difference, dose calculation, and daily CBCT localization. The evaluation confirms that the method of generating S‐CT image is clinically acceptable, and can be reliably implemented.

A previous study^(14)^ has shown that using multi‐atlas‐based DIR in S‐CT production would produce ‘smooth’ effect in the S‐CT image. The effect could reduce the gray scale contrast and lead to less attenuation for radiation beam. In addition, using atlas‐DIR method directly suffers the uncertainty caused by interpatient registration and would produce blur in high‐contrast areas. Subsequently, it will result in inaccurate treatment localization. Our proposed image intensity selection method has the ability to overcome these drawbacks via selecting proper atlas‐CT for different voxel in S‐CT determined using the intersection of the atlas‐ROI masks. Consequently, the proposed method achieved much better result compared with the direct arithmetic mean method.^12^, ^13^ Figures 3(c) and (d) demonstrate a typical case and the absolute error histogram comparison. The white spot (Figs. 3(c) and (d)) in the bladder of S‐CT was caused by contrast inside bladder of two patients in the atlas library. However, that does not affect the results of dose calculation or CBCT localization.

A question of practical importance is how many atlases need to be used? As shown on Fig. 2(b), autosegmentation accuracy increases slightly as more atlases are used. However, the computational cost increases linearly with the number of atlas for both autosegmentation and S‐CT generation. Optimal atlas number was not addressed in this study. Additionally, in order to generate an S‐CT image that shares the same ROI structures with subject MR image, we introduced manual modification for the resulting autosegmentation of soft‐tissue ROIs. However, the effort of manual correction depends on the MR segmentation tool. The robustness of the proposed S‐CT generation algorithm or the sensitivity to manual modification needs to be investigated in a future study.

Technical feasibility to include the S‐CT image in clinical workflow with respect to treatment planning and CBCT image‐guided localization is another important issue for MRI‐alone treatment simulation.^(6)^ The proposed method is clinically feasible, and the total time for generating an S‐CT using our proposed method was 255∼30 min using nine paired atlas images (185∼20 min for multi‐atlas DIR, 75∼10 min for S‐CT construction).

## V. CONCLUSIONS

An approach of creating an S‐CT image from a MR simulation image was developed and evaluated. The approach is clinically feasible and acceptable for both treatment planning and CBCT image‐guided localization of prostate cancer treatment. It has a potential to be extended to the other treatment sites.

## ACKNOWLEDGMENTS

This work has been partially supported by Elekta R&D grant, and the research planning license provided by Philips.

## COPYRIGHT

This work is licensed under a Creative Commons Attribution 4.0 International License.

## Supporting information

Supplementary Material

Supplementary Material

Supplementary Material
