# Supplementary figures and images for: MR image‐based synthetic CT for IMRT prostate treatment planning and CBCT image‐guided localization
Source: J Appl Clin Med Phys. 2016 May 8;17(3):236–45. doi: 10.1120/jacmp.v17i3.6065 (PMC5690904; doi:10.1120/jacmp.v17i3.6065)

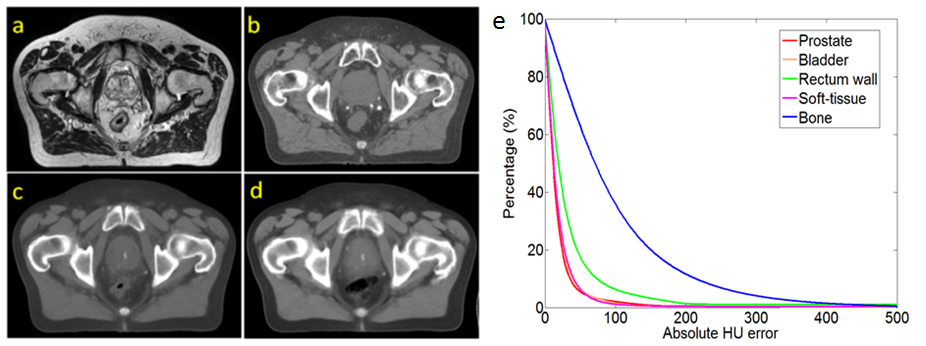

Supplement: Supplementary file 1 — Supplementary Material [file ACM2-17-236-s001.png]

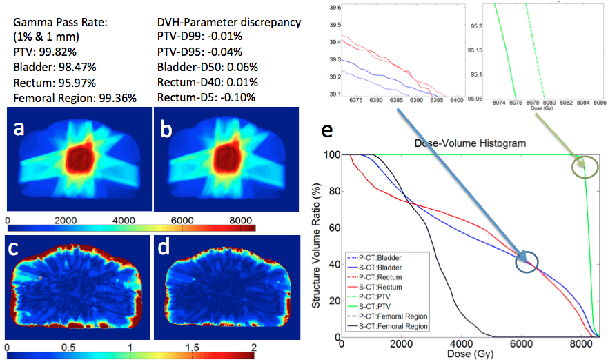

Supplement: Supplementary file 2 — Supplementary Material [file ACM2-17-236-s002.png]

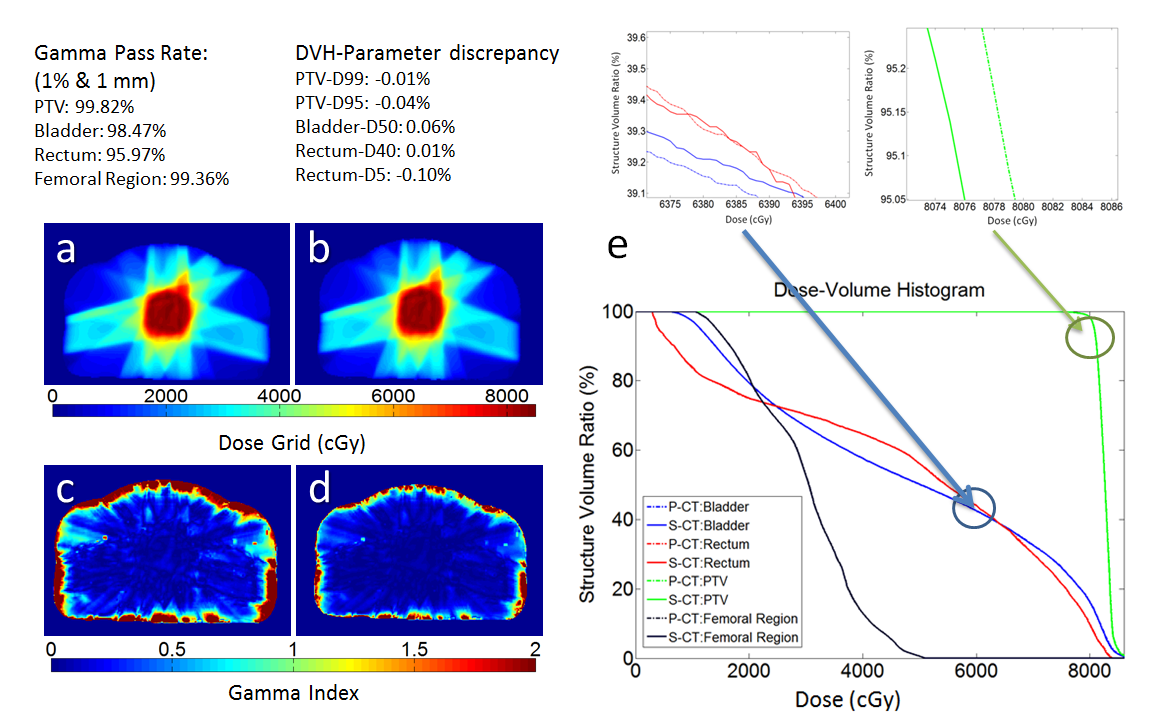

Supplement: Supplementary file 3 — Supplementary Material [file ACM2-17-236-s003.png]
